# Vitamin D deficiency and risk of acute lung injury in severe sepsis and severe trauma: a case-control study

**DOI:** 10.1186/2110-5820-4-5

**Published:** 2014-02-24

**Authors:** Nicolas Barnett, Zhiguo Zhao, Tatsuki Koyama, David R Janz, Chen-Yu Wang, Addison K May, Gordon R Bernard, Lorraine B Ware

**Affiliations:** 1 Division of Allergy, Pulmonary and Critical Care Medicine in the Department of Medicine, Vanderbilt University, T1218 MCN, 1161 21st Avenue South, Nashville, TN, USA; 2 Department of Biostatistics, Vanderbilt University, Nashville, TN, USA; 3 Division of Trauma and Surgical Critical Care, Department of Surgery, Vanderbilt University Medical Center, Nashville, TN, USA; 4 Division of Internal Medicine Taichung Veterans General Hospital, Taichung, Taiwan; 5 Department of Pathology, Microbiology and Immunology, Vanderbilt University, Nashville, TN, USA

**Keywords:** Vitamin D, Sepsis, Trauma, Acute lung injury, Critical illness

## Abstract

**Background:**

The aim of this study was to determine the association between 25-hydroxyvitamin D (25-OHD) levels at the onset of critical illness and the development of acute lung injury/acute respiratory distress syndrome (ALI/ARDS) in patients with sepsis or trauma.

**Methods:**

We performed two nested case-control studies of 478 patients with sepsis and trauma with or without ALI/ARDS admitted to the medical, surgical and trauma ICUs of a tertiary-care center. Cases consisted of patients with either sepsis or trauma and ALI/ARDS; controls consisted of equivalent numbers of matched patients with either sepsis or trauma alone. We measured serum 25-OHD levels the morning after ICU admission and used multivariable regression to assess the relationship between 25-OHD and diagnosis of ALI/ARDS during the first four ICU days, controlling for age, gender, diabetes, smoking status and season.

**Results:**

25-OHD levels did not differ between cases with ALI/ARDS and controls in either the sepsis or trauma cohorts. Using a conditional logistic regression model, sepsis patients during the winter season with higher 25-OHD levels were more likely to develop acute lung injury (odds ratio 1.68, 95% confidence interval of 1.05 to 2.69, *P* = 0.03). This association did not hold for the trauma cohort in either season. Sepsis and trauma patients had a lower risk of hospital mortality at higher 25-OHD levels but neither relationship reached significance. Higher one-year mortality after trauma was associated with lower 25-OHD levels (HR 0.50, CI 0.35,0.72 *P* = 0.001).

**Conclusions:**

Serum 25-OHD measured early after admission to intensive care is not associated with the development of acute lung injury, hospital or one-year mortality in critically ill patients with sepsis although lower 25-OHD levels were associated with higher one-year mortality in patients with severe trauma.

## Background

Epidemiologic and biologic evidence supports a pivotal role for vitamin D in health and disease [1-9]. Serum vitamin D levels (as reflected by the serum concentration of 25-OHD) in the lowest quartile (< 45 nmol/L) are associated with excess all-cause mortality in the general population. In a meta-analysis of randomized controlled trials in which vitamin D was administered for any health condition, supplementation with standard doses reduced overall mortality [10], an observation substantiated by a recent Cochrane review [11]. Biologic data reinforce these observations [9]. Specifically, vitamin D and its receptor exhibit key anti-inflammatory [12], membrane-stabilizing [13] and anti-microbial properties [14] at barrier sites such as the gut [15], lung [16] and skin [17].

In critical illness, vitamin D deficiency is prevalent, [18,19] has been associated with increased length of ICU stay [20] and adverse outcomes [21]. Low 25-OHD levels measured within the year prior to intensive care admission [22] but also at critical care initiation [23] are significantly associated with increased short and long term mortality. Acute lung injury (ALI) and acute respiratory distress syndrome (ARDS) are syndromes of acute respiratory failure occurring in the setting of common clinical conditions including sepsis and trauma. It is still not understood why some but not all patients at risk develop ALI/ARDS [24]. Several features of the pathogenesis of ARDS, including the central role of innate immunity, inflammation and the presence of increased lung endothelial and epithelial permeability [25], suggest a candidate role for vitamin D in modulating its development. Vitamin D’s anti-inflammatory properties [26], pervasive role in endothelial [27] and epithelial barrier functions [16] and potential to augment innate immunity [14] make it an important avenue to explore in the pathogenesis of ALI.

We hypothesized that lower 25-OHD levels at the onset of critical illness would be associated with the development of ALI/ARDS in two distinct populations at high risk for ALI/ARDS: patients with severe sepsis and patients with severe trauma. To test this hypothesis we measured serum 25-OHD levels early in the course of critical illness in two nested, parallel, case-control studies: patients with severe sepsis with or without ALI/ARDS; and patients with severe trauma with or without ALI/ARDS.

## Methods

### Study design and patients

Cases and controls were selected from the Validating Acute Lung Injury biomarkers for diagnosis (VALID) study. VALID is a single-center 2,500 patient observational cohort study of critically ill patients at high-risk for ALI/ARDS [28,29], enrolling since 2006. Patients were recruited from medical, surgical, cardiac and trauma ICUs at Vanderbilt University Medical Center. All ICU patients mechanically ventilated > 12 hours were eligible for enrollment. Patients were excluded if they were < 18 years of age, were in any other ICU for > three days, had cardiac arrest before enrollment, or if they were expected to be transferred out of ICU by day two. Patients were also excluded if they were admitted for an uncomplicated overdose, routine postoperative admission after cardiothoracic surgery or if they had a history of severe chronic lung disease. Comprehensive clinical data were obtained including the Acute Physiology and Chronic Health Evaluation II (APACHE II) score [30], Injury Severity Score (ISS) [31], daily phenotyping for sepsis [32], organ failure [33] and ALI/ARDS using the American European Consensus Conference definitions [34]. Severe trauma was defined as an ISS score > 15. Non-pulmonary organ failures were assessed by Brussels scoring [33]. Informed consent was obtained from an available surrogate or the participants themselves when possible. A waiver of consent was also granted due to the minimal risk for study participants. The Institutional Review Board at Vanderbilt approved the study protocol.

For the sepsis study, cases were defined as patients with severe sepsis at the time of enrollment meeting consensus criteria for ALI/ARDS during the first four days in the ICU after enrollment. Controls were patients with severe sepsis at the time of enrollment not meeting consensus criteria for ALI/ARDS during the first four days in the ICU. For the trauma study, cases were defined as patients with severe trauma who met consensus criteria for ALI/ARDS during the first four days in the ICU. Controls were patients with severe trauma who did not meet consensus criteria for ALI/ARDS during the first four days in the ICU. In both studies, controls were matched one to one to cases for severity of illness using the APACHE II for the sepsis cohort and the ISS for the trauma cohort. Cases and controls were also matched for age (within five years), length of daylight at enrollment (within one hour) and ethnicity - all factors that may affect 25-OHD levels [35]. We identified 480 suitable patients from VALID: 120 cases with ALI/ARDS and sepsis, 120 cases with ALI/ARDS and trauma and a matching number of sepsis (120) and trauma (120) controls. One patient from the trauma cohort had no serum - its matched pair was therefore removed leaving a total of 478 eligible patients (119 trauma cases and 119 trauma controls). Of these, one trauma patient and one sepsis patient did not have sufficient serum for vitamin D analysis (see Additional file 1).

### Vitamin D measurement

Samples were obtained the morning after ICU admission, processed within one hour of collection, centrifuged and stored at -80°C until further analysis. For quantitative determination of 25-OHD we used a Food and Drugs Administration approved [35], direct, competitive chemiluminescence immunoassay (CLIA) using the DiaSorin LIASON 25-OHD total assay (Heartland Assays, Ames, IA, USA) [36]. Serum 25-OHD was measured in duplicate.

### Definition of deficiency

Sufficiency was defined as 25-OHD > 75 nmol/L, insufficiency 50 to 75 nmol/L and deficiency < 50 nmol/L [37].

### Exposure, covariates and outcomes

The primary exposure variable was the serum 25-OHD level measured on the morning of ICU day two. Multiple covariates were selected *a priori* including age, gender, diabetes, smoking status and season for their potential confounding effects on risk of ALI and modulation of vitamin D levels. Levels of 25-OHD in the tables are presented in nmol/L.

The primary outcome was development of ALI/ARDS in the first four days of admission to the ICU, which was the time frame for intensive clinical phenotyping for ALI/ARDS in the VALID study. Hospital mortality and all-cause one-year mortality were pre-specified secondary end-points.

### Statistical analysis

We fit multivariable conditional logistic regression models using log transformed 25-OHD to investigate the effects of 25-OHD on our primary and secondary outcomes. Pre-specified exploratory variables included age, gender, smoking status, diabetes and season. As an additional analysis, we *a priori* stratified patients into two seasons, a summer season (21 June to 21 Dec) and a winter season (21 Dec to 21 June) and thus included an interaction term for season in our model to investigate the season-specific effects on our primary outcome of interest (ALI/ARDS).

We used Cox proportional hazard models to investigate the effects of 25-OHD on the secondary outcomes - hospital mortality and one-year mortality. In the trauma cohort, ISS was missing for 34 subjects. A multiple imputation method with ten imputation datasets was used to accurately estimate the parameters and standard errors of risk factors. All statistical inferences were assessed at a two-sided 5% significant level. All summary statistics, graphics, and models were generated using R version 2.13.1 statistical software.

In the absence of pilot data, we relied on published data in determining appropriate sample size for this study [19,38]. Based on a reconstructed distribution with mean 35 to 38 nmol/L and a standard deviation of approximately 22, a sample of size of 240 (120 cases and 120 controls) has a power of 90% (with type I error rate of 5%) to detect a difference equivalent to approximately 40% to 50% of a within-group standard deviation using a simple *t*-test. Additionally, with a total sample size of approximately 240 for each cohort, we included five variables in our multivariable model without risk of over-fitting. To determine the association of 25-OHD levels with our primary (ALI/ARDS) and secondary (hospital and one-year mortality) outcomes in our regression models, we opted to compare 25-OHD levels at fixed points on a continuous scale: third (upper) and first (lower) quartiles.

## Results

### Sepsis cohort

**Figure 1:**
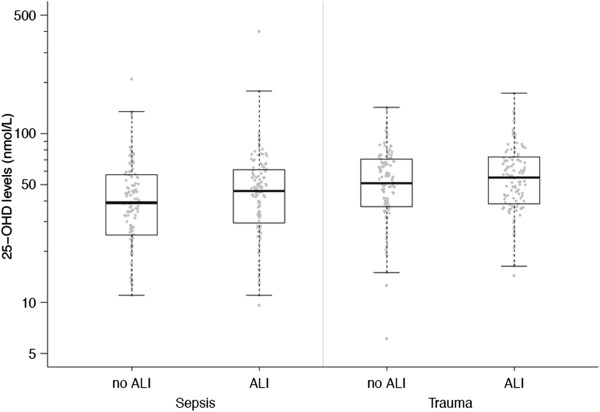
**Box plot summary of 25-hydroxyvitamin D (25-OHD) levels by cohort and case-control status.** Twenty-five-OHD levels were not significantly different between patients with or without acute lung injury/acute respiratory distress syndrome (ALI/ARDS). Horizontal bars represent medians, boxes encompass the 25^th^ to 75^th^ percentile and error bars encompass the 10^th^ to 90^th^ percentile.

Patient characteristics and vitamin D status are summarized in Table 1. Cases and controls were well matched for baseline characteristics but differed on other important characteristics such as presence of diabetes, use of minute ventilation and risk factors for ALI/ARDS (Table 1). Eighty-eight percent of patients with severe sepsis had vitamin D deficiency or insufficiency (serum 25-OHD < 75 nmol/L). Median serum 25-OHD concentration was 43.2 nmol/L. There was no significant difference in serum 25-OHD levels between ALI cases and controls (median 45.8 versus 39.0 nmol/L, *P* = 0.12) (Table 1 and Figure 1).

**Table 1:** Baseline characteristics of patients from the sepsis cohort

**Characteristic**	**N**	**Cases**	**Controls**	**Combined**	***P*-value**
		**(n = 120)**	**(n = 120)**	**(n = 240)**	
Age^c^ (IQR)	240	59.3 (53.2, 65.7)	59.3 (52.7, 65.7)	59.3 (53.2, 65.7)	0.885^a^
Female gender (%)	240	53 (44)	56 (47)	109 (45)	0.791^b^
Smoking (%)	231				0.505^b^
Never		40 (34)	33 (29)	73 (32)	
Former		44 (38)	40 (35)	84 (36)	
Current		33 (28)	41 (36)	74 (32)	
Diabetes (%)	240	31 (26)	47 (39)	78 (32)	0.045^b^
Summer season (%)	240	34 (28)	35 (29)	69 (29)	1^b^
Day length hours (IQR)^c^	240	12.0 (10.6, 13.6)	12.0 (10.6, 13.5)	12.0 (10.6, 13.6)	0.655^a^
APACHE II Score (IQR)^c^	240	28 (23, 32.2)	28 (23, 32.2)	28 (23, 32.2)	NA
Number of non-pulmonary organ failures (%)	240				0.753^a^
0		15 (12)	24 (20)	39 (16)	
1		60 (50)	45 (38)	105 (44)	
2		39 (32)	39 (32)	78 (32)	
3		4 (3)	9 (8)	13 (5)	
4		2 (2)	3 (2)	5 (2)	
Days of ICU stay (IQR)	240	9 (6,15)	6 (4,10)	7 (4,14)	0.008^a^
Hospital mortality (%)	240	30 (25)	31 (26)	61 (25)	1^b^
One-year mortality (%)	240	66 (55)	71 (59)	137 (57)	0.614^b^
Risk factors for ALI/ARDS	240				< 0.001^b^
Non-pulmonary sepsis		55 (46)	70 (60)	125 (53)	
Pneumonia		39 (33)	22 (19)	61 (26)	
Aspiration		24 (20)	8 (7)	32 (14)	
Other or None^d^		1 (1)	16 (14)	17 (16)	
Patients on ventilator		89 (74)	71 (59)	160 (67)	0.016^b^
25-OHD (nmol/L) (IQR)	239	45.8 (29.9, 60.8)	39.0 (25.0, 56.9)	43.2 (27.6, 58.8)	0.123^a^
Vitamin D status (%)	239				0.358^b^
Deficiency (< 50 nmol/L)		72 (60)	82 (69)	154 (64)	
Insufficiency (50 to 75 nmol/L)		32 (27)	26 (22)	58 (24)	
Sufficiency (> 75 nmol/L)		16 (13)	11 (9)	27 (11)	

Data given as median with interquartile range (IQR) or frequency with (%) of patients.

N is the number of non-missing values.

^a^Wilcoxon signed rank test; ^b^McNemar’s test; ^c^Matched; ^d^Other or None (pancreatitis, multiple transfusions, trauma).

**Figure 2:**
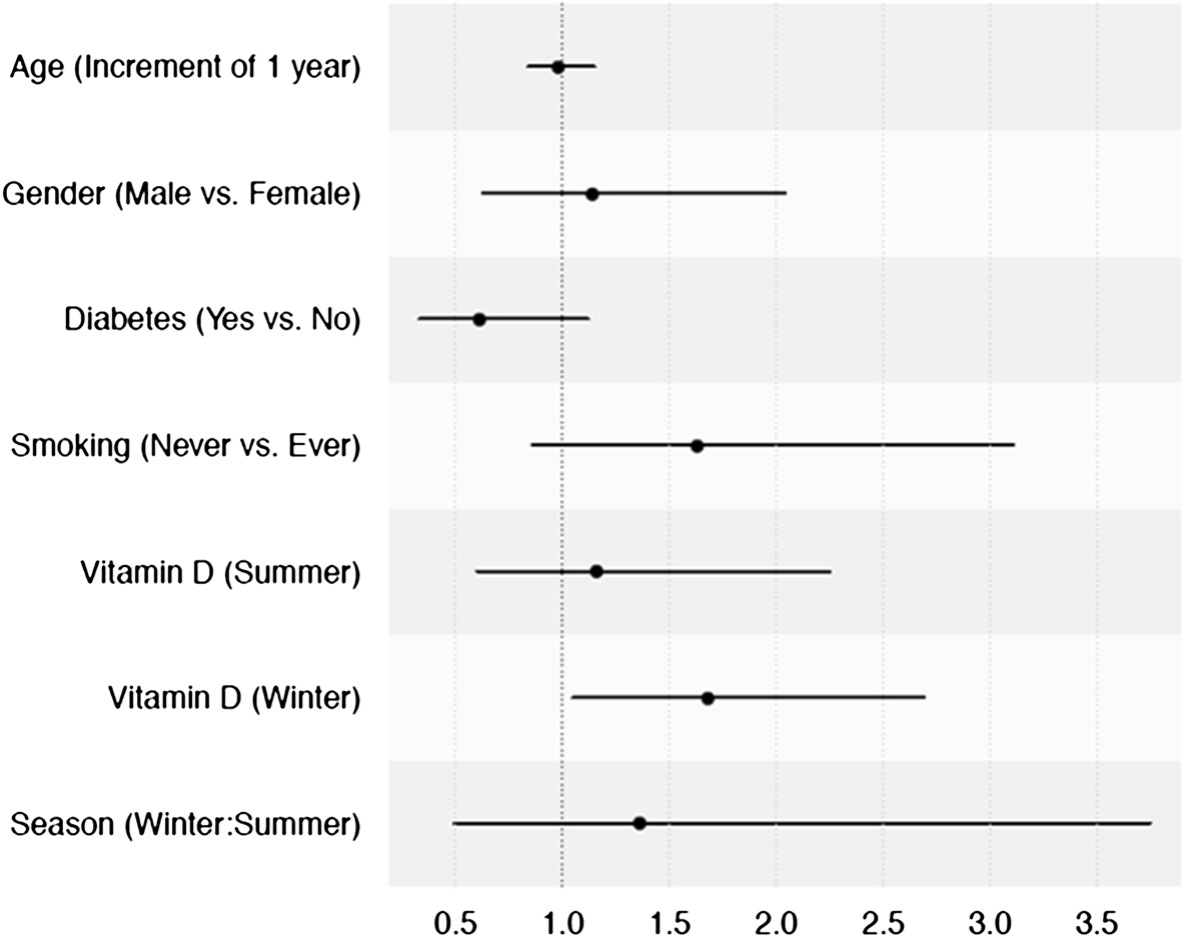
**Forest plot summary of logistic regression analysis for risk of acute lung injury/acute respiratory distress syndrome (ALI/ARDS) in the sepsis cohort.** Higher vitamin D levels in winter were associated with a higher odds ratio (OR) for developing ALI/ARDS: OR 1.68 (95% CI 1.054 to 2.69), (*P* = 0.03). OR (black dots) and the corresponding CIs (black lines) for the risk of ALI/ARDS for each covariate are shown.

A higher serum level of 25-OHD in patients admitted during winter months was associated with developing ALI/ARDS; odds ratio (OR) was 1.68 (95% CI 1.054 to 2.69), (*P* = 0.03) for the upper (58.3 nmol/L) compared to the lower quartile (27.6 nmol/L). During summer months we observed no association between 25-OHD levels and development of ALI/ARDS; OR 1.16 (95% CI 0.589 to 2.25), (*P* = 0.66) (Table 2 and Figure 2).

**Table 2:** Conditional logistic regression analysis for risk of acute lung injury/acute respiratory distress syndrome (ALI/ARDS) in the sepsis cohort (left) and trauma cohort (right)

**Characteristics**	**Sepsis cohort**			**Trauma cohort**		
	**OR**	**95% CI**	***P*-value**	**OR**	**95% CI**	***P*-value**
Age (increment of one year)	0.98	0.84 to 1.15	0.805	0.93	0.77 to 1.13	0.482
Gender (male versus female)	1.14	0.63 to 2.04	0.668	0.81	0.39 to 1.71	0.586
Diabetes (yes versus no)	0.61	0.33 to 1.12	0.110	1.10	0.39 to 3.08	0.854
Smoking (never versus ever)	1.63	0.86 to 3.11	0.136	1.15	0.54 to 2.41	0.720
Vitamin D levels (summer)^a^	1.16	0.60 to 2.25	0.663	1.25	0.55 to 2.86	0.591
Vitamin D levels (winter)^a^	1.68	1.05 to 2.69	0.029	1.23	0.74 to 2.06	0.425
Season (winter versus summer)^b^	1.36	0.49 to 3.75	0.553	0.61	0.20 to 1.86	0.385

*Abbreviations:* OR odds ratio, CI confidence interval.

^a^third versus first quartile; sepsis cohort: 58.9 versus 27.6 nmol/L; trauma cohort: 71.9 versus 37.2 nmol/L.

^b^vitamin D was fixed at median; sepsis cohort: 43.2 nmol/L; trauma cohort: 53.4 nmol/L.

When all patients with severe sepsis were considered together (both cases and controls) patients with 25-OHD levels at the highest quartile (58.3 nmol/L) had a hazard ratio of 0.74 (95% CI 0.53 to 1.05) for hospital mortality compared to the lowest quartile (27.6 nmol/L), a result that was not significant (*P* = 0.09) (Table 3). Patient survival time up to one year was also not associated with 25-OHD levels (*P* = 0.24) (Table 4).

**Table 3:** Survival analysis for hospital mortality in the sepsis cohort (left) and the trauma cohort (right)

**Characteristics**	**Sepsis cohort**			**Trauma cohort**		
	**HR**	**95% CI**	***P*-value**	**HR**	**95% CI**	***P*-value**
Age (increment of one year)	1.02	1.00 to 1.05	0.100	1.04	1.01 to 1.08	0.019
Gender (male versus female)	1.19	0.67 to 2.12	0.552	1.16	0.37 to 3.62	0.794
Diabetes (yes versus no)	1.14	0.64 to 2.02	0.663	0.82	0.22 to 3.05	0.768
Smoking (never versus ever)	0.82	0.44 to 1.54	0.543	0.90	0.31 to 2.66	0.855
Vitamin D levels^a^	0.74	0.53 to 1.05	0.092	0.59	0.30 to 1.19	0.140
Season (winter versus summer)	0.86	0.48 to 1.54	0.618	0.85	0.27 to 2.73	0.787

*Abbreviations:* HR hazard ratio, CI confidence interval.

^a^third versus first quartile; sepsis cohort: 58.9 versus 27.6 nmol/L; trauma cohort: 71.9 versus 37.2 nmol/L.

**Table 4:** Survival analysis for delayed, one-year mortality in the sepsis cohort (left) and the trauma cohort (right)

**Characteristics**	**Sepsis cohort**			**Trauma cohort**		
	**HR**	**95% CI**	***P*-value**	**HR**	**95% CI**	***P*-value**
Age (increment of one year)	1.02	1.00 to 1.04	0.023	1.06	1.04 to 1.08	0.023
Gender (male versus female)	1.26	0.90 to 1.78	0.181	2.04	1.00 to 4.18	0.051
Diabetes (yes versus no)	1.04	0.72 to 1.49	0.841	0.63	0.28 to 1.45	0.280
Apache II/ISS score^b^	1.35	1.05 to 1.73	0.019	1.04	0.68 to 1.60	0.849
Vitamin D levels (high versus low)^a^	0.87	0.68 to 1.10	0.240	0.50	0.35 to 0.72	< 0.001

*Abbreviations:* HR hazard ratio, CI confidence interval.

^a^third versus first quartile; sepsis cohort: 58.9 versus 27.6 nmol/L; trauma cohort: 71.9 versus 37.2 nmol/L.

^b^third versus first quartile; sepsis cohort: Apache II score, 32.2 versus 23; trauma cohort: ISS score, 36.0 versus 23.5.

### Trauma cohort

**Figure 3:**
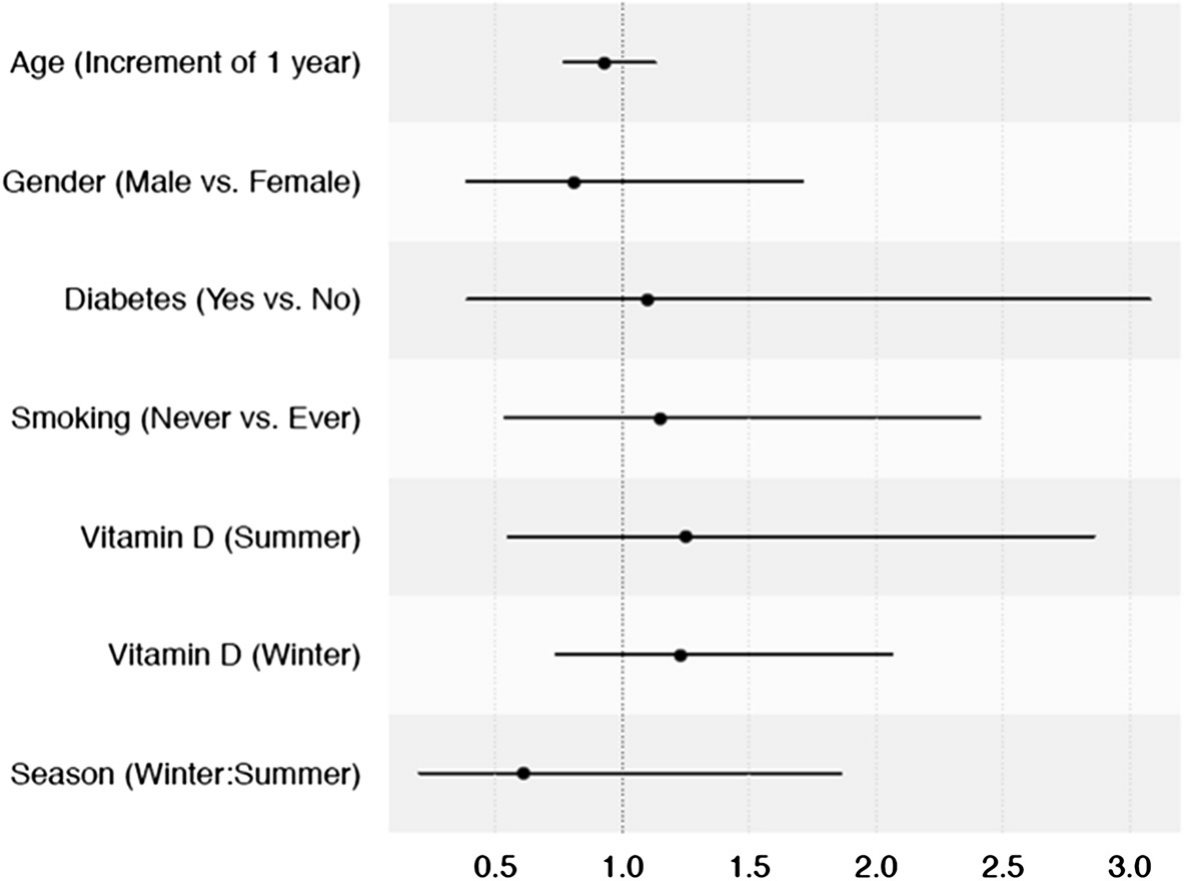
**Forest plot of logistic regression analysis for risk of acute lung injury/acute respiratory distress syndrome (ALI/ARDS) in the trauma cohort.** Vitamin D levels were not associated with risk of ALI/ARDS in severe trauma. Odds ratio (black dots) and the corresponding confidence intervals (black lines) for the risk of ALI/ARDS for each covariate are shown.

Trauma patient characteristics are summarized in Table 5. The trauma cohort was well matched for baseline and secondary characteristics. The median serum 25-OHD level in trauma patients was 53.4 nmol/L. Eighty-one percent of trauma patients were either vitamin D insufficient or deficient. There was no significant difference in serum 25-OHD levels between trauma ALI and non-ALI groups (54.9 versus 50.8 nmol/L, *P* = 0.30) (Figure 1) nor was any statistically significant relationship detected on multivariable analysis (Table 2 and Figure 3).

**Table 5:** Basline characteristics of patients from the trauma cohort

**Characteristic**	**N**	**Cases**	**Controls**	**Combined**	***P*-value**
		**(n = 119)**	**(n = 119)**	**(n = 238)**	
Age^c^	238	44.7 (30.8, 58.2)	46.8 (29.8, 56.8)	46.2 (30.4, 57.8)	0.651^a^
Female gender (%)	238	35 (29)	33 (28)	68 (29)	0.883^b^
Smoking (%)	194				0.370^b^
Never		36 (35)	30 (33)	66 (34)	
Former		8 (8)	3 (3)	11 (6)	
Current		60 (58)	57 (63)	117 (60)	
Diabetes (%)	238	16 (13)	16 (13)	32 (13)	1^b^
Summer season (%)	238	48 (40)	48 (40)	96 (40)	1^b^
Day length hours (IQR)^c^	238	12.6 (10.8, 13.8)	12.6 (10.7, 13.9)	12.6 (10.8, 13.9)	0.331^a^
APACHE II score (IQR)	238	26.0 (21.5, 29.5)	24.0 (19.0, 27.5)	25.0 (20.0, 29.0)	0.078^a^
ISS^c^ (IQR)	204	29.0 (24.0, 35.8)	29.0 (22.0, 36.0)	29.0 (23.5, 36.0)	0.993^a^
Number of non-pulmonary organ failures (%)	238				0.645^a^
0		42 (35)	49 (41)	91 (38)	
1		61 (51)	52 (44)	113 (47)	
2		13 (11)	15 (13)	28 (12)	
3		3 (3)	3 (3)	6 (3)	
Days of ICU stay (IQR)	238	11.0 (7.0, 18.5)	9.0 (5.0, 16.0)	10.0 (6.0, 18.0)	0.155^a^
Hospital mortality (%)	238	10 (8)	13 (11)	23 (10)	0.663^b^
One-year mortality (%)	238	20 (17)	25 (21)	45 (19)	0.511^b^
Risk factors for ALI/ARDS	235				
Severe trauma		116 (99)	116 (99)	232 (99)	
Aspiration		2 (2)	0 (0)	2 (1)	
Other or None^d^		0 (0)	1 (1)	1 (1)	
Patients on ventilator		115 (97)	115 (97)	230 (97)	1^b^
25-OHD (nmol/L) (IQR)	237	54.9 (38.3, 72.5)	50.8 (36.9, 69.8)	53.4 (37.2, 71.9)	0.302^a^
Vitamin D Status (%)	237				0.606^b^
Deficiency (< 50 nmol/L)		54 (45)	59 (50)	113 (48)	
Insufficiency (50 to 75 nmol/L)		37 (31)	38 (32)	75 (32)	
Sufficiency (> 75 nmol/L)		28 (24)	21 (18)	49 (21)	

Data given as median and interquartile range (IQR) or frequency with (%) of patients.

N is the number of non-missing values.

^a^Wilcoxon signed rank test; ^b^McNemar’s test; ^c^Matched; ^d^Other or None (multiple transfusions).

In the trauma cohort, 25-OHD levels were also not significantly associated with time to hospital mortality - hazard ratio 0.59 (95% CI 0.3 to 1.20), (*P* = 0.14) (Table 3). On the other hand, 25-OHD levels significantly predicted all-cause, one-year mortality from trauma (*P* = 0.001), hazard ratio 0.5 (95% CI 0.35 to 0.72) (Table 4.) Age was also significantly associated with hospital and all-cause mortality from trauma with hazard ratios of 1.07 (95% CI 1.01 to 1.08), (*P* = 0.02) and 1.06 (95% CI 1.04 to 1.08, *P* < 0.001) respectively. All other variables had no significant association with hospital or one-year mortality in our models.

## Discussion

In this case-control study of two ‘*at risk’* populations for ALI/ARDS, patients with severe sepsis or severe trauma, vitamin D levels measured on the morning after ICU admission were not associated with development of ALI/ARDS. Furthermore, in a stratified analysis of patients with sepsis during the winter season, higher rather than lower vitamin D levels were associated with increased risk for ALI/ARDS (*P* = 0.03). The biologic rationale for this finding is uncertain. One potential explanation is that vitamin D physiology is complex. Higher vitamin D levels may, in certain situations, paradoxically increase the pathogen burden [39] leading to a worsening of pro-inflammatory stress in the lung and thence to ALI/ARDS. Another, more likely, interpretation for our primary findings is that low levels of vitamin D are not associated with development of ALI/ARDS and are potentially epiphenomena of severity and/or chronicity of illness [40]. In the current study, when cases and controls were rigorously matched for severity of illness using APACHE II scores and other clinical characteristics, no association between ALI/ARDS and 25-OHD levels was apparent. This result was consistent across two diverse patient groups and risk factors for ALI/ARDS: severe sepsis and severe trauma.

Experimental evidence for the deleterious effects of vitamin D deficiency on organ and immune dysfunction abounds [13,41] but evidence in the intensive care arena is lacking. Jeng *et al*. [19] found that both septic and non-septic ICU patients had low vitamin D levels without any demonstrable differences in levels between groups suggesting that critical illness *per se* is preceded by or results in low vitamin D levels. On the other hand, Braun *et al*. [22] in a much larger 2,399 patient cohort provide evidence for an association of vitamin D deficiency with mortality in the ICU. They showed an association of ICU, hospital, 30, 60, 90 and 365-day mortality in patients with the lowest levels of pre-admission (up to one year prior to admission) vitamin D levels. A subsequent study by the same group [23] examined peri-admission (seven days prior to and/or seven days following ICU admission) 25-OHD levels, and likewise reported a positive association between 25-OHD deficiency and 30-day mortality. Secondary end-points of 90 and 365-day mortality also were associated with peri-admission vitamin D deficiency.

In this study, sepsis patients with increased vitamin D levels demonstrated a non-significant trend to reduced hospital mortality (*P* = 0.09) that did not persist to one-year (*P* = 0.24). By comparison, Braun *et al*. [22] obtained 25-OHD samples up to a year prior to ICU admission potentially deterring from their predictive power at the time of critical illness. To dispel this limitation a follow-up study [23] examined 25-OHD levels around the time of critical care initiation and observed a similar association. However, the time span from measurement to critical care initiation was relatively large (14 days) as opposed to the narrower 36 hours defined in our study. Moreover, neither analysis included information on important co-variates that may affect 25-OHD levels such as smoking or aggregate scores of chronic disease and physiologic perturbation such as APACHE II score that may affect mortality risk [30]. In the current study we elected to control for severity of illness because it is likely to impact negatively both on vitamin D levels and mortality. In many patients, relative malnutrition and periods of physical inactivity precede critical illness. Second, a dilutional effect may occur owing to vigorous fluid resuscitation at the time of critical illness [42]. While we did not control for volume of resuscitation in our analysis, matching by severity of illness (APACHE II or ISS scores) should imply a similar resuscitation burden between and across cohorts, thereby minimizing the potentially confounding effects of acute fluid loading.

Interestingly, lower 25-OHD levels were significantly associated with one-year mortality (*P* < 0.001) in trauma patients, the first time this association has been observed. Very little has been published regarding long term outcomes from major trauma. A recent study implicated age older than 65, hospitalization greater than 28 days and unintentional falls as risk factors for delayed one-year mortality following trauma [43]. Our data also identified older age to be a significant risk factor for delayed mortality after trauma (Table 4). Older age and unintentional falls could plausibly be biologically linked to lower 25-OHD levels though the potential for residual confounding remains. For example, confounding by BMI, for which we do not have data in our study, is a possible explanation for this result. A higher BMI correlates both with worse outcomes [44] from trauma and is associated with lower 25-OHD levels [45]. However, cases and controls for this study were matched with our primary end-point (ALI/ARDS) in mind and not mortality and as such this result is a novel and potentially hypothesis-generating observation.

The study has some limitations. Study design allowed us to control for severity of illness but the study was not powered for mortality analysis and the case-control design may limit the generalizability of the findings. A second limitation is that we did not measure serial vitamin D levels and therefore could not correlate vitamin D kinetics during critical illness to acute decompensations or improvements in clinical status. Higgins *et al*. [46] recently showed a mean reduction in 25-OHD levels during critical illness of 2.6 mmol/L by day three and 6.0 mmol/L by day ten. Reid *et al*. [47] similarly observed a mean diminution of 2.7 mmol/L in 25-OHD levels 48 hours after an acute surgical insult for elective hip or knee surgery together with a mean reciprocal increase in CRP from 5 to 116 mg/ml - a situation that may pertain to all fat-soluble vitamins [48]. They conclude that 25-OHD is likely a negative acute phase reactant with low levels persisting for several months after an inflammatory insult.

A third potential limitation is that we were unable to control for vitamin D binding protein (VDBP). Falls in this protein during critical illness could impact on 25-OHD levels. However, the association of vitamin D with its binding protein is complex [49] and non-linear. Furthermore, higher levels of VDBP may limit the bioavailability of the free and bioactive vitamin [50]. Because of this complexity, future studies of vitamin D deficiency and outcomes in ICU may wish to measure both serial vitamin D levels and VDBP.

Additionally, we were also unable to control for pre-hospital use of calcium and vitamin D that could be a potential confounder of serum 25-OHD levels. However, this information can be difficult to obtain even prospectively in critically ill patients and hence is absent from the majority of studies of vitamin D in critical illness [19,22,23]. We have found only one small study with information on calcium/vitamin D supplementation [38]. In this study, only 13% of the total cohort was on supplements but 7% had end-stage renal failure, a patient group commonly on supplementation. Moreover, supplementation in this study was predictive of vitamin D deficiency supporting the hypothesis that most patients on supplements had concomitant renal failure with hypovitaminosis D. In addition, with a median age in the mid 40s, it is unlikely that patients in our trauma cohort were on vitamin D supplementation. Finally, we did not obtain data and, therefore, could not control for transfusion requirements, which may influence ARDS risk [51] and potentially be associated with vitamin D levels. There is however no evidence on the stability of fat-soluble vitamins in transfused blood and matching by trauma severity (ISS) would plausibly suggest similar transfusion schedules.

Our study has several strengths. To our knowledge, it is the first study to investigate the association of ALI/ARDS in critical illness with 25-OHD levels. We report a consistent lack of association between lower 25-OHD levels and risk of ALI/ARDS across two cohorts of patients who were carefully matched for age, gender, smoking status and severity of illness with distinct risks for ALI/ARDS. Both cohorts mirror each other in suggesting that patients with ALI/ARDS have slightly elevated 25-OHD levels in comparison to their control counterparts lending internal validity to the findings. The study population is broad, rigorously phenotyped and representative of a critical care population in terms of disease acuity (based on APACHE II and ISS scores), basic demographics, the spectrum of ICUs (cardiac, medical, surgical and trauma) and hospital mortality across both cohorts. Furthermore, blood was sampled at clear and uniform time-points, early in the course of critical illness allowing for a consistent analysis of the relationship between the exposure, 25-OHD levels, and the outcome of interest, ALI/ARDS.

Although the findings of this study are not definitive, they do call into question the hypothesis that vitamin D supplementation might help reconstitute immune, endothelial, epithelial and thence organ function across a variety of clinical and biological end-points [52]. Indeed, the Institute of Medicine’s recent report [53] stated that there was insufficient evidence at present to recommend higher dietary intake of vitamin D other than for bone health. Prospective studies of supplementation in critical illness are awaited before any definitive conclusions can be drawn. However recent negative supplementation studies in respiratory [54,55] and cardiovascular disease [56] tend to support the Institute of Medicine’s position.

## Conclusions

This case-control study of 478 patients with sepsis or trauma showed no association between lower 25-OHD levels and development of ALI/ARDS in stringently matched cases and controls. Contrary to our initial hypothesis, we noted the unexpected finding of higher 25-OHD levels associated with ALI/ARDS in winter in the sepsis cohort. In a secondary analysis, we discerned no associations between lower 25-OHD levels and hospital or delayed mortality with the exception of an association of higher 25-OHD levels with a lower risk of one-year mortality from trauma. While the present study does not preclude a potential beneficial effect of vitamin D supplementation in severe sepsis, our findings suggest that any potential benefit is not likely to be related to prevention of ALI/ARDS; a hitherto unsuspected mortality benefit may, however, exist for patients with severe trauma.

## Abbreviations

25-OHD: 25-hydroxyvitamin D; ALI/ARDS: acute lung injury/acute respiratory distress syndrome; APACHE II: Acute Physiology and Chronic Health Evaluation; CLIA: competitive chemiluminescence immunoassay; ISS: Injury Severity Score; VALID: Validating Acute Lung Injury biomarkers for Diagnosis study; VDBP: vitamin D binding protein.

## Competing interests

The authors declare that they have no competing interests.

## Authors’ contributions

NB designed the study, analyzed the data and wrote and edited the manuscript. ZZ and TK analyzed the data and edited the manuscript. DJ and CYW analyzed patient data and edited the manuscript. AM enrolled patients and edited the manuscript. GB designed the study and edited the manuscript. LW conceived and designed the study, analyzed data and edited the manuscript. All authors read and approved the final manuscript.

## Supplementary Material

Additional file 1 Flow chart of patients in study. Click here for file
