# Supplementary figures and images for: Vitamin D deficiency and risk of acute lung injury in severe sepsis and severe trauma: a case-control study
Source: Ann Intensive Care. 2014 Feb 24;4:5. doi: 10.1186/2110-5820-4-5 (PMC3944729; doi:10.1186/2110-5820-4-5)

Appendix 1. **Flow chart of patients in study.**

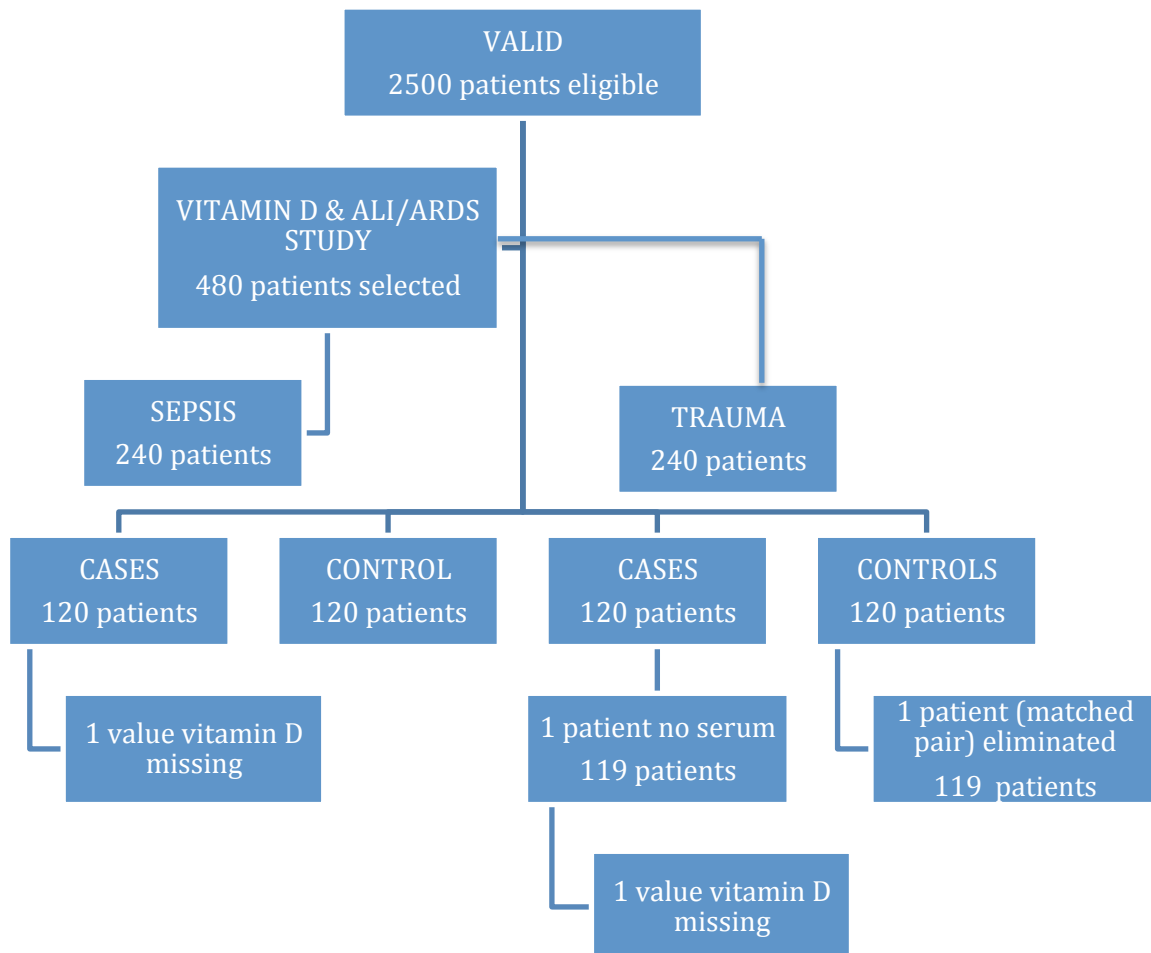

Supplement: Additional file 1 — Flow chart of patients in study. [file 2110-5820-4-5-S1.pdf]
